# Supplementary material for: The potential of resilience indicators to anticipate infectious disease outbreaks, a systematic review and guide
Source: PLOS Glob Public Health. 2023 Oct 10;3(10):e0002253. doi: 10.1371/journal.pgph.0002253 (PMC10564242; doi:10.1371/journal.pgph.0002253)
Supplement: Attachment — Submitted filename: rebuttal_letter.pdf [file pgph.0002253.s005.pdf]

Dr. Catherine Kyobutungi, Professor Madhukar Pai

Editor-in-Chief

Journal PLOS Global Public Health 05-04-2023

Rebuttal letter of systematic review titled 'The potential of resilience indicators to anticipate infectious disease outbreaks, a systematic review and guide'

Dear Editors,

We thank the editorial staff for their generous comments on the previous version of the manuscript, previously submitted under the reference PGPH-D-22-01707.

Clara Delecroix,

On behalf of all authors.

Reviewer's comment:

"Specifically, we are concerned that the literature search and, more importantly, data extraction were only performed by one person. To ensure that literature searches and the data extraction procedure are thorough and rigorous, these tasks should be completed by two (or more) independent reviewers, with a consensus procedure in place for disagreements between reviewers."

We have now addressed this comment. The literature search has been performed by two reviewers independently. This process led to 47 (5%) disagreements during the title/abstract selection process, and 5 (8%) disagreements during the full-text selection process. Disagreements were discussed at the end of each round until a consensus was reached. The final corpus of included studies was not altered by the additional reviewer. This process is described in the Material and methods section of the manuscript, lines 161-164:

"The first selection was made based on the title and abstract. We retrieved 60 publications in this round. The second round of selection was based on full-text, using the selection criteria (Table 1), retrieving a final 37 publications (Fig 2, S3 Table). Both rounds of selection were performed by two reviewers independently (CD, RR). At the end of each round, disagreements were discussed with both reviewers until a consensus was reached."

Additionally, the data extraction has been performed by two reviewers independently. Reviewers disagreed on 44 entries (11%). Disagreements were resolved at the end of the process by discussing with all reviewers. This process is described in the Material and methods section of the manuscript, lines 181-183.

"The data were extracted by two reviewers independently (CD, IAL, EHN). Disagreements were discussed with all reviewers at the end of the information retrieval process until an agreement was reached."

These additional steps did not change the results and conclusions of our study.
