# Supplementary material for: The potential of resilience indicators to anticipate infectious disease outbreaks, a systematic review and guide
Source: PLOS Glob Public Health. 2023 Oct 10;3(10):e0002253. doi: 10.1371/journal.pgph.0002253 (PMC10564242; doi:10.1371/journal.pgph.0002253)
Supplement: Attachment — Submitted filename: response_to_reviewers.docx [file pgph.0002253.s006.docx]

Dr. Reuben Kiggundu

Academic Editor

Journal PLOS Global Public Health	30-06-2023

**Rebuttal letter of systematic review titled ‘The potential of resilience indicators to anticipate infectious disease outbreaks, a systematic review and guide’**

Dear Editors,

We thank the reviewers for their generous comments on the previous version of the manuscript. We have now addressed their concerns in the manuscript. In particular, we have added a paragraph in the discussion to include potential data sources suitable for the use of resilience indicators.

Clara Delecroix,

On behalf of all authors.

Reviewer #1’s comments:

*The study is well narrated and elaborated. Currently the issue of infectious disease outbreak needs to be revised and new method should be developed. So that combination approaches should be practiced. So, this study will help in this area.*

Thank you for taking your time to review our manuscript, and for your encouraging words.

*In line 299 re-emergence of malaria is not in all places please specify the country.*

We have now addressed this comment.

Lines 298-300: *“The anticipation of vector-borne disease transitions using resilience indicators was shown in three studies investigating dengue in Puerto Rico (25), plague in Madagascar (25), and malaria re-emergence in Kenya (56), and lymphatic filariasis elimination (55)*.”

Reviewer #2’s comments:

*The manuscript is well written and organized. Data is a correctly available and schemes are clear.*

Thank you for reviewing our manuscript and for your kind words.

*It would be interesting to include some evaluation on the different health/surveillance systems of countries, considering the data provided by each system can be a limitation to resilience factors. If the evaluation is not possible, I suggest including as a limitation in the article.*

Thank you for your suggestion. We now included a discussion on potential data sources in the discussion as well as a short discussion on the potential use of surveillance systems for influenza and covid-19.

Lines 460-479: “*Few countries have surveillance systems able to achieve such high-quality data, especially since sufficient data prior to the start of an outbreak is necessary. In general, we can distinguish active surveillance efforts, sentinel surveillance, and passive surveillance. Active surveillance, namely the active seeking of cases of a given disease in a population, allows to estimate the prevalence of a disease with high accuracy, but is extremely costly and not achievable on the long-term. Sentinel surveillance system, i.e., the monitoring of disease prevalence in a population via a network of general practitioners, can help achieve consistent, good quality data assuming that these are reported with a sufficient temporal resolution and that the system is sufficiently sensitive (40). Covid-19 and influenza are examples of diseases that are monitored via sentinel surveillance. Influenza is monitored through sentinel surveillance systems within the GISRS initiative from WHO, and cases are reported with a weekly resolution in the FluID database (60). Similarly, covid-19 is nowadays consistently monitored via sentinel surveillance systems and sometimes reported in publicly available databases (61–64). However, the quality of sentinel surveillance systems depends on the health-seeking behavior and access to health-care facilities in the population. Similarly, the quality of data provided by passive surveillance systems is context-dependent. Most included case studies relied on data obtained from passive surveillance systems in areas with sufficient access to healthcare facilities and for diseases with sufficient symptomatic cases. However, such systems can underestimate the prevalence in periods of low transmission (65,66) and hamper the prediction performance of resilience indicators if the reporting rate increases together with the prevalence of the disease (41). Low income countries with circulating diseases of poverty such as cholera or Ebola as well as neglected tropical diseases lack a constant surveillance system in place prior to outbreaks and the surveillance is mostly reactive, making the data unsuitable for resilience indicators (67–70).”*
